# Altered Self‐Referential‐Related Brain Regions in Depersonalization‐Derealization Disorder

**DOI:** 10.1002/brb3.70314

**Published:** 2025-02-11

**Authors:** Yuan Jia, Nan Song, Yanzhe Ning, Hong Zhu, Linrui Dong, Sitong Feng, Hongxiao Jia, Mingkang Song, Sisi Zheng

**Affiliations:** ^1^ The National Clinical Research Center for Mental Disorders & Beijing Key Laboratory of Mental Disorders, Beijing Anding Hospital Capital Medical University Beijing China; ^2^ Department of Science and Technology, Department of Psychology Bournemouth University Poole UK; ^3^ Xiamen Xianyue Hospital, Xianyue Hospital Affiliated with Xiamen Medical College, Fujian Psychiatric Center Fujian Clinical Research Center for Mental Disorders Xiamen China; ^4^ Advanced Innovation Center for Human Brain Protection Capital Medical University Beijing China

**Keywords:** depersonalization, derealization, rest‐state functional magnetic resonance, self‐referential

## Abstract

**Objective:**

We aimed to explore the alteration in topology and network properties in self‐referential‐related brain regions of individuals with depersonalization–derealization disorders (DPD), using evidence from resting‐state functional magnetic resonance imaging (rs‐fMRI).

**Methods:**

We first determined the regions of interest (ROIs) using Neurosynth, based on which we conducted an ROI‐wise functional connectivity search to create a self‐referential‐related network and performed a topographical analysis. We then compared the analyzed properties from the rs‐fMRI of disordered individuals to those of healthy controls to generate differential properties, based on which we conducted a machine learning‐based disease diagnostic model.

**Results:**

The study found significant changes in connectivity between brain regions associated with self‐referential processing in individuals with DPD compared to healthy controls. Correlation analysis showed negative correlations between “unreality of surroundings” and connectivity between the left inferior frontal gyrus (IFG) pars orbitalis and left insula and between “perceptual alterations” and connectivity between the left pregenual and subgenual anterior cingulate cortex (ACC). Graph theoretical analysis revealed increased local and global efficiency but decreased characteristic path length. The accuracy of the classification model was 0.885, and the area under the curve was 0.928.

**Conclusions:**

Individuals with DPD showed alterations in brain topography and changes in network properties within self‐referential‐related brain regions; specifically, the changes in cortical midline structures and insula could be related to the underlying mechanism of DPD, highlighting potential targets for future research and therapeutic strategies.

## Graphical abstract

This study used resting‐state fMRI to identify changes in self‐referential network and brain topology in depersonalization–derealization (DPD) patients, highlighting the anterior cingulate cortex, medial prefrontal cortex, and insula as key regions. The high accuracy of the machine‐learning model suggests these changes could be biomarkers for DPD diagnosis.

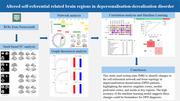

## 1 Introduction

According to the American Psychiatric Association (2013), depersonalization–derealization disorder (DPD) is a condition whereby individuals experience a sense of detachment from both their surroundings and the self. While DPD affects approximately 1% of the general population, the percentage is higher in psychiatric inpatients, ranging from 17.5%–41.9%, compared to 5%–20% in psychiatric outpatients (J. Yang et al. 2022). Individuals with DPD report symptomatic feelings such as living in a dream‐like state, no longer existing, or a sense of nothing being real (Hunter et al. 2017). Despite these symptoms, however, individuals with DPD are known to have intact reality‐testing capabilities, as demonstrated by the frequent use of “as if…” phrases in their descriptions of feelings (Hunter et al. 2017).

Self‐referential processing is the processing of stimuli experienced as closely related to the self (Northoff et al. 2006). Cortical midline structures (CMS) and the default mode network (including the medial prefrontal cortex [mPFC], posterior cingulate cortex [PCC], and anterior cingulate cortex [ACC]) are considered central to this process (Davey et al. 2016; H. Kim 2012; Northoff et al. 2006). Other regions that may be critically involved are the insula and the dorsolateral prefrontal cortex (DLPFC) (Northoff and Bermpohl 2004; Qin and Northoff 2011). Specifically, the mPFC may be involved in the meta‐representation of stimulus that is required to evaluate the stimulus as self‐referential (Gusnard et al. 2001; K. Kim and Johnson 2014; Northoff and Bermpohl 2004); the PCC is associated with self‐reflection and autobiographical memory retrieval (Ferris et al. 2024; Northoff and Bermpohl 2004; Philippi et al. 2015; Rolls 2019); the ACC is involved in the monitoring of self‐referential stimuli (Carter et al. 2001; Northoff and Bermpohl 2004; J. Yang et al. 2016); the insula is related to the processing of interoceptive stimuli and interoceptive awareness ((Bud) Craig 2009; Kuehn et al. 2016; Qin and Northoff 2011).

The relationship between the symptoms of DPD and impaired self‐referential processing is evidenced by recent behavioral and neuroimaging studies, which suggest that impaired self‐referential processing may be a pathogenic mechanism for DPD. For example, Ketay et al. (2014) found that participants with DPD exhibited greater activation in the right ACC, bilateral mPFC, and left middle frontal gyrus (MFG) when processing self‐faces versus stranger's faces. Moreover, they also found that the severity of DPD is significantly correlated with the activation of mPFC and the left MFG. Furthermore, our prior study (Zheng et al. 2022) indicated that the mPFC, which participates in the self‐referential processing mechanism (Frewen et al. 2020), is also more directly involved in the mechanisms of DPD. Another of our studies utilizing a subliminal self‐face recognition paradigm demonstrated alterations in self‐face recognition in individuals with DPD, suggesting a potential impairment of self‐referential processing (Liu et al. 2022). Besides being correlated to previously mentioned regions (ACC, mPFC, MFG), DPD is suggested to be related to insula activity, which contributes to interoceptive awareness (i.e., our ability to sense ourselves) (Craig 2002). For example, increased insula activity is associated with clinical improvements in DPD symptoms, whereas reduced insula activity is associated with attenuation of emotional experience (Medford et al. 2016). These studies have all suggested that the self‐referential processing‐related regions are involved in the mechanism of DPD.

Regardless of these previous findings regarding deficits in self‐referential processing and corresponding changes in specific brain regions of individuals with DPD, it remains unclear whether these individuals exhibit abnormal brain networks or topology, especially in those regions related to self‐referential processing. To investigate this problem, we established regions of interest (ROIs) related to self‐referential processing using meta‐analysis, where the ROIs represent the self‐referential processing‐related brain regions. We then conducted seed‐based functional connectivity (FC) analysis to find the altered brain regions that are in communication with the self‐referential‐processing‐related brain regions. ROI–ROI FC analysis (i.e., network analysis) was conducted to find the altered self‐referential processing‐related network, and graph theoretical analysis was conducted to find the modified organization in the self‐referential network.

## 2 Methods

### 2.1 Participants and Clinical Assessment

The sample size was estimated using G*Power 3.1.9.7. We performed a prior power analysis (*F* tests, ANOVA fixed effects, omnibus, one‐way). We targeted 80% statistical power, with a medium effect size of *f* = 0.25 and significance level *α* = 0.05, and we got a total sample size of 128 with two groups. Finally, 66 right‐handed DPD patients were recruited from the Outpatient Department of Beijing Anding Hospital, Capital Medical University, from January 2020 to December 2022. All examinations were carried out under the guidance of the Declaration of Helsinki. Sixty‐six healthy control (HC) participants were recruited as a control group to match the samples in the DPD group. The present study was approved by the Ethics Committee of Beijing Anding Hospital, Capital Medical University, China (No. 2020‐17). Informed consent was taken from all the participants present in the study.

All DPD patients were diagnosed according to the criteria of the 10th revision of the International Classification of Diseases (World Health Organization 1992) and screened according to the Chinese version of the Dissociative Disorders Interview Schedule (Vencio et al. 2019) and the Mini‐International Neuropsychiatric Interview (MINI). Specific inclusion criteria for the DPD group include (a) age 15–45 years old. This range captures the peak onset of depersonalization disorder and ensures neuroplasticity while excluding potential neurodevelopmental confounds, (b) right‐hand dominant (controlled for potential neuroanatomical variations associated with handedness), and (c) a score of 70 or above on the Cambridge Depersonalization Scale (CDS) (Sierra and Berrios 2000). Specific exclusion criteria for the DPD group include (a) transient experiences of depersonalization or derealization (or both) due to trauma, fatigue, or substance use; (b) with other psychiatric comorbidities; (c) a history of neurological disorders or family history of hereditary neurological disorders; (d) history of substance addiction or brain trauma; (e) gross morphological anomalies, as evidenced by brain MRI; and (f) any electronic or metal implants. Patients with past medication history were documented but not automatically excluded.

We also recruited 66 HC participants who matched in age, gender, and education level from the community and college. These HC participants were determined to be free of previous DPD symptoms or psychiatric diseases based on the MINI screening process and the two‐item CDS interviews (Hunter et al. 2017). All HC participants were also screened for histories of acute or chronic illness and drug allergy. All HCs were right‐handed.

### 2.2 MRI Data Acquisition

Resting‐state functional magnetic resonance imaging (fMRI) data were acquired using a 3.0 Tesla MRI scanner (Prisma 3.0; Siemens, Germany) in the Beijing Anding Hospital, Capital Medical University, China. fMRI data were acquired with a single‐shot, gradient‐recalled echo‐planar imaging sequence with the following parameters: repetition time = 2000  ms, echo time = 30 ms, flip angle = 90°, matrix = 64 × 64, field of view (FOV) = 200 mm × 200  mm, slice thickness = 3.5 mm, gap = 1 mm, 33 axial sections, and 240 volumes.

High‐resolution brain structural images were acquired with a T1‐weighted three‐dimensional (3D) multi‐echo magnetization‐prepared rapid gradient‐echo (MPRAGE) sequence (echo time: 3.39 ms, repetition time: 2530 ms, slice thickness: 1.3 mm, voxel size: 1.3 × 1 × 1 mm^3^, FOV: 256 × 256 mm^2^, and volume number: 128).

Each participant was given a 30‐min rest period before scanning to ensure consistency, and participants were instructed to remain still and stay awake during scanning. Foam head holders were used to immobilize the participants during scanning.

### 2.3 Image Preprocessing

We opted for using the DPABISurf toolbox for all image preprocessing (Yan et al. 2021) due to its ability to generate surface‐based and volume‐based analysis based on surface‐space and volume‐space results. We used the default preprocessing pipeline within the DPABISurf toolbox, including converting the user‐specified data into Brain Imaging Data Structure (BIDS), skull‐stripping, spatial normalization, brain tissue segmentation, surface reconstruction for T1‐weighted images and slice‐timing correction, realignment, head‐motion estimation, spatial registration for functional images, band‐pass temporal filtering (0.01–0.1 Hz), and spatial smoothing using a 6 mm full‐width half‐maximum Gaussian kernel. The detailed methods were described in the article by Yan et al. (2021) and Esteban et al. (2019). It is worth noting that the smoothing step was not performed before the graph theoretical analysis (suggested by Alakörkkö et al. 2017) since the level of spatial smoothing affects the degrees and other centrality measures of functional network nodes.

### 2.4 Seed‐Based FC Analysis

First, to perform FC analysis, we established ROIs using the coordinates from the self‐referential meta‐analysis performed by the Neurosynth platform (Yarkoni et al. 2011), which is a web‐based platform for automated and large‐scale synthesis of functional neuroimaging research. Neurosynth used advanced text‐mining techniques to automatically extract and analyze results from thousands of published neuroimaging studies. The detailed steps were described by Yarkoni et al. (2011). This automated approach achieved a similar level of sensitivity and scope compared to the more effort‐intensive approaches used in previous studies while significantly reducing the time and labor required for meta‐analysis.

We selected 166 studies for our meta‐analysis (see Table S1 for a complete list of studies) and identified the self‐referential‐related brain regions using the uniformity test map. To achieve a more detailed meta‐analysis, we used the meta‐analysis results from the Neurosynth platform in our study, which provided a map of the meta‐analysis results rather than several coordinates. This *p* value for the false discovery rate (FDR) was adjusted to 0.05. Next, the coordinates with peak *z*‐scores and all clusters larger than 50 voxels were identified by the xjview toolbox (http://www.alivelearn.net/xjview/). Furthermore, the spherical masks with a radius of 6 mm were generated using the MarsBaR toolbox (Brett et al. 2002).

Subsequently, we conducted a seed‐based FC analysis. In brief, the residual BOLD time course average was extracted from the masks, and Pearson's correlation coefficients were computed between whole brain voxels (both in surface and volume space). Finally, the coefficients were transformed into *z*‐scores to increase normality. Thus, first‐level FC maps were generated for both surface and volume space.

### 2.5 Network and Graph Theoretical Analysis

To establish a self‐referential network, we used the automated anatomical labelling atlas 3 (AAL3) (Rolls et al. 2020) to separate the large clusters from the meta‐analysis results into different labeled brain regions (voxels > 20). Then, we created 6 mm radius spherical masks with the MarsBar toolbox using the peak coordinates of each separated cluster. Next, we overlapped the masks with the results from the meta‐analysis so that the final masks consisted of only voxels from the meta‐analysis result. Finally, we used the masks to extract BOLD signals and performed FC analysis between these ROIs to create the first‐level self‐referential network.

Furthermore, based on the constructed weighted brain networks, we examined the topological organization changes of small‐world metrics and local and global efficiency. The small‐world metrics mainly included the clustering coefficient (*C*_p_), characteristic path length (*L*_p_), their normalized versions using 100 random networks (lambda and gamma), and small‐worldness (sigma). The range of sparsity was from 0.01 to 0.5, and the step of sparsity was 0.01. For each metric, the area under the curve (AUC) for a smaller range of density (0.1–0.34, step of 0.01) was calculated, similar to previous studies (Wang et al. 2017; H. Yang et al. 2021).

### 2.6 Group‐Level Analysis

Two‐sample *t* tests were used to compare neuroimaging values between the two groups, where sex, age, education level, and head motion (mean framewise displacement) were included as covariates. Cohen's ƒ^2^ values were used to describe the effect sizes. There were several ROIs in the FC analysis part; therefore, we used a family‐wise error (FWE; 1000 simulations, vertex *p* threshold = 0.001) to correct for surface‐based analysis and a Gaussian random field (GRF; voxel *p* threshold = 0.001) for volume‐based analysis at cluster *p* with 0.05/*N*, where *N* represents the number of ROIs. For network analysis, we opted for the network‐based statistic (NBS) approach (5000 permutations, edge *p* < 0.01, component *p* < 0.05). In the graph theoretical analysis part, the significant *p* value cutoff was determined by permutation testing with an FDR < 0.05 (5000 permutations) (Winkler et al. 2016).

We then performed a Spearman correlation analysis between CDS‐29 scores and fMRI measures and constructed a classification model using JASP (Version 0.16.4) (JASP Team 2023). Afterward, we conducted the *k*‐nearest neighbors (KNN) (Laaksonen and Oja 1996) classification with the feature selected from the network analysis and graph theoretical analysis (train and validation data:test data = 8:2, seed = 300, weights = optimal, distance = Manhattan, max.neareast neighbors = 38); specifically, we used the features that were different between DPD individuals and HCs. We estimated the model with Leave‐One‐Out Cross‐Validation (LOOCV) (Wong 2015); all subsequent claims are based on these LOOCV results.

## 3 Results

### 3.1 Clinical Information

A total of 66 DPD individuals and 66 HC participants were selected for this study. Overall, the power of the sample size was calculated using G*power 3.197 (post hoc analysis) and a power > 0.95 was found. The detailed parameters were as follows: Test family: *t* tests, Statistical test: linear multiple regression: fixed model, single regression coefficient, Effect size range: 0.1–0.13. Therefore, the power indicated that the sample size of this study is sufficient.

In terms of gender, age, or education level, the two groups did not differ significantly (all *p *> 0.05; Table 1). Table 1 shows the CDS‐2 scores of patients with DPD.

**TABLE 1:** Clinical information of DPD patients and HCs.

	DDD (*N* = 66)	HC (*N* = 66)	*p*
Gender (male/female)	45/21	41/24	0.5837
Age	23.3 (5.1)	21.9 (4.2)	0.086
Education (years)	13.5 (2.9)	14.3 (2.8)	0.112
Mediation (untreated/treated)	45/21		
Course (years)	4.61 (4.02)		
CDS total score	166.35 (49.33)		
Numbing	32.55 (15.69)		
Unreality of self	39.80 (14.45)		
Perceptual alterations	20.02 (12.25)		
Unreality of surroundings	14.38 (5.56)		
Temporal disintegration	21.91 (10.15)		

*Note*: Values shown are mean (SD)/number, statistics, and *p* value of two‐sample *t* tests (chi‐square test for gender) comparing patients with DPD and healthy controls.

Abbreviation: CDS, Cambridge Depersonalization Scale; DPD, depersonalization–derealization disorders; HC, healthy controls.

### 3.2 Seed‐Based FC Analysis

**FIGURE 1:**
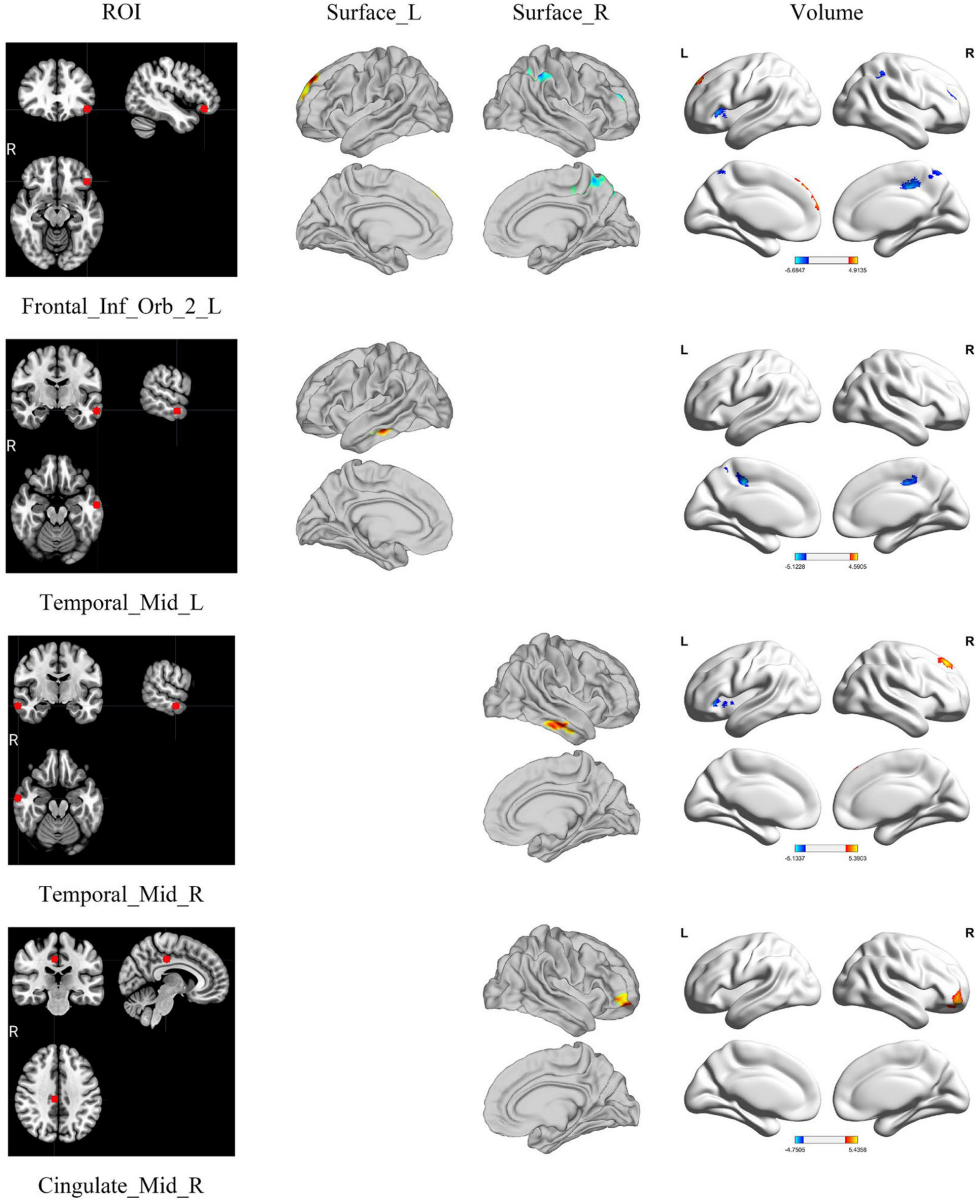
The results of seed‐based FC analysis.

We used the 12 ROIs established from the meta‐analysis (Figure S1; Table S2) to conduct FC analysis. Figure 1 and Table 2 show the FC analysis results. With multiple comparison corrections, the seed‐based FC analysis of the left IFG pars orbitalis (IFGorb), left middle temporal gyrus (MTG), right MTG, and the right middle cingulate and paracingulate gyri (MCC) remained significant. Based on left IFGorb and right MCC as ROIs, FC results were consistent for both volume and surface analyses.

**TABLE 2:** The results of seed‐based FC analysis.

ROILabel	Space	Cluster size	MNI (*x*, *y*, *z*)	Intensity	Brain region
Frontal_Inf_Orb_2_L	Volume	554	−11.5, 49.5, 47.5	4.77	SFG, L
	Volume	501	16.5, −64.5, 69.5	−4.51174	Precuneus, R
	Volume	388	36.5, 51.5, 29.5	−5.68468	MFG, R
	Volume	381	8.5, −34.5, 47.5	−5.19435	MCC, R
	Volume	346	48.5, −44.5, 45.5	−4.66244	IPG, R
	Volume	295	−35.5, 23.5, 1.5	−4.93368	Insula, L
	Surface	56	−11.4, 47.2, 39.0	4.74954	SFG, L
	Surface	245	8.7, −31.6, 40.1	−5.04053	MCC, R
Temporal_Mid_L	Volume	281	8.5, −32.5, 49.5	−5.1228	MCC, R
	Volume	275	−11.5, −28.5, 45.5	−5.09994	MCC, L
	Surface	35	−56.3, −29.8, −17.5	5.29943	ITG, L
Temporal_Mid_R	Volume	427	20.5, 37.5, 49.5	5.39025	SFG, R
	Volume	353	−47.5, 11.5, −4.5	−4.76146	Insula, L
	Surface	58	60.5, −16.5, −22.2	4.82434	ITG, R
Cingulate_Mid_R	Volume	547	36.5, 53.5, −12.5	5.43582	MFG, R
	Surface	60	35.9, 48.6, −9.4	4.93365	MFG, R

*Note*: The cluster size in volume‐based analysis represents voxels, while in surface‐based analysis represents vertices.

Abbreviations: IPG, inferior parietal gyrus; ITG, interior temporal gyrus; L, left; MCC, middle cingulate; MFG, middle frontal gyrus; R, right; SFG, superior frontal gyrus.

### 3.3 Network Analysis Results

**FIGURE 2:**
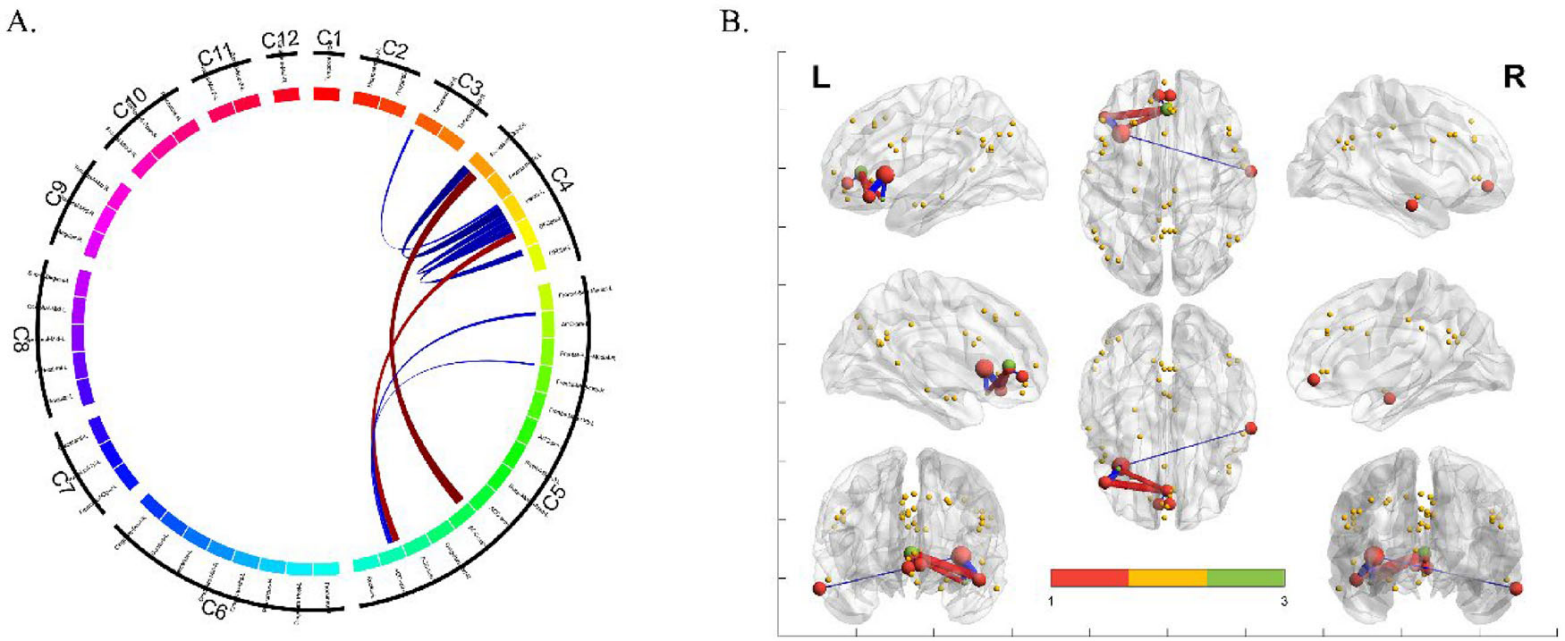
Result of network analysis. (A) The circos of 49 × 49 network results. (B) The BrainNet Viewer results.

Figure 2 shows the results of the network analysis. Compared to those of HC participants, the connections in the individuals with DPD significantly decreased: between left insula and right MTG, between left insula and left IFGorb, between left posterior orbital gyrus (OFC) and left insula, between left lateral OFC and left insula, between left subgenual of ACC and pregenual of ACC, and between medial orbital of superior frontal gyrus (SFG) and left subgenual of ACC. The significantly increased connections were as follows: between the left orbital part of IFG and the left supracallosal of ACC and between the left subgenual of ACC and the left posterior OFC.

### 3.4 Graph Theoretical Analysis

**FIGURE 3:**
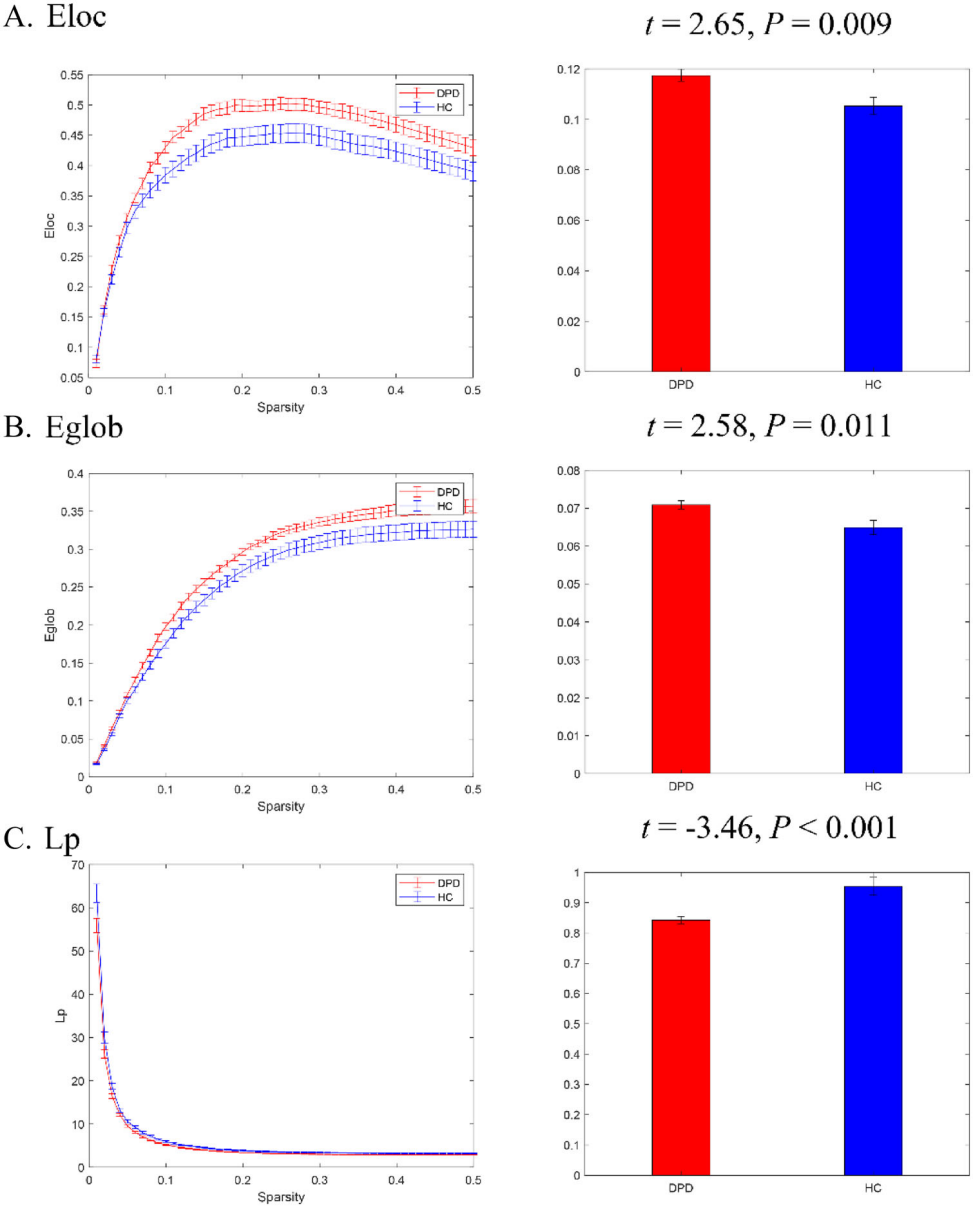
Results of graph theoretical analysis. Eglob, global efficiency; Eloc, local efficiency; Lp, characteristic path length.

Compared to HC participants, the local and global efficiency of individuals with DPD have significantly increased, while LP significantly decreased (Figure 3). Other properties of graph theory indicators were not different from HCs.

### 3.5 Correlation Analysis and Machine Learning

**FIGURE 4:**
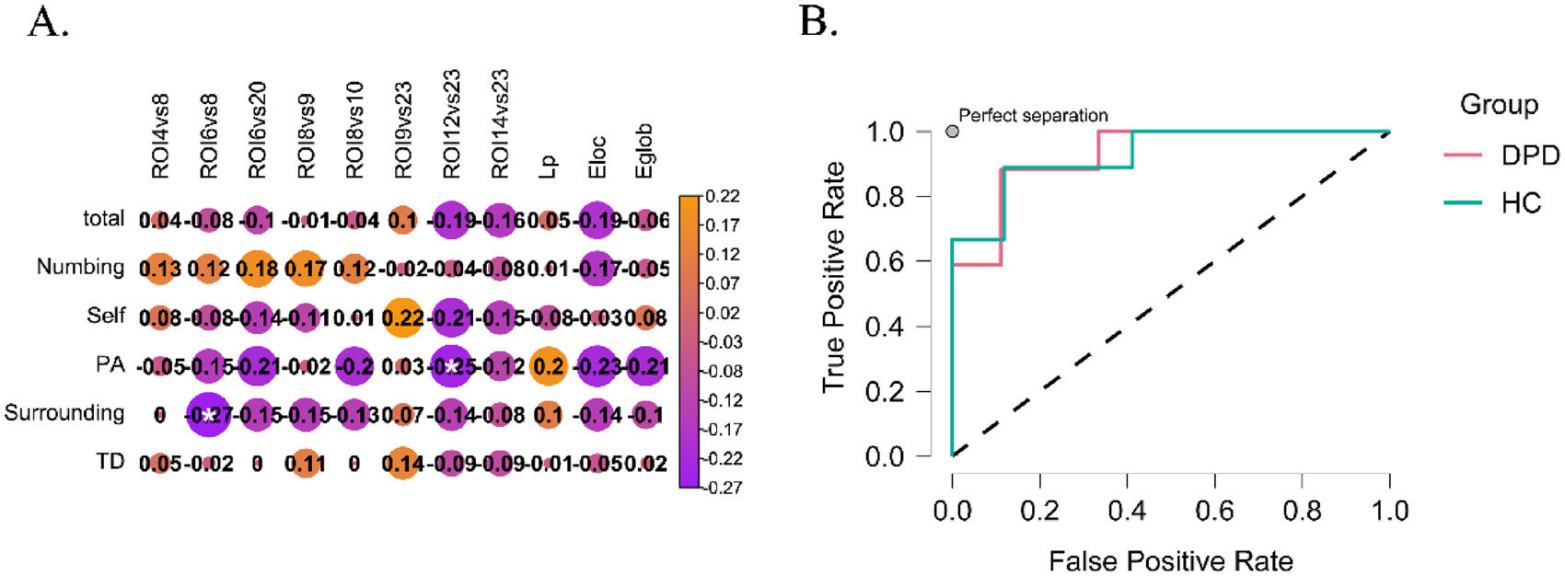
Correlation analysis and machine learning results. (A) Correlation analysis. The detail information of the variables: total, CDS total scores; Self, the unreality of delf factor scores of CDS; PA, the perceptual alterations factor scores of CDS; Surrounding, the unreality of surroundings factor score of CDS, TD, the temporal disintegration factor scores of CDS; ROI4, Temporal_Mid_R, the right middle temporal gyrus; ROI6, Frontal_Inf_Orb_2_L, left IFG pars orbitalis; ROI8, Insula_L; ROI9, OFCpost_L, left posterior orbital gyrus; ROI10, OFClat_L, lateral orbital gyrus; ROI12, ACC_pre_L, left anterior cingulate cortex, pregenual; ROI14, Frontal_Med_Orb_R, right superior frontal gyrus, medial orbital; ROI20, ACC_sup_L, left anterior cingulate cortex, supracallosal; ROI23, ACC_sub_L, left anterior cingulate cortex, subgenual. (B) ROC analysis of machine learning.

The unreality of surroundings factor scores of CDS‐29 was negatively correlated with the connectivity between the left IFG pars orbitalis and left insula; the perceptual alterations factor scores of CDS‐29 were negatively correlated with the connectivity between the left pregenual of ACC and left subgenual part of the ACC (Figure 4A).

The variables used in the correlation analysis were selected to conduct the KNN model. Figure 4B shows the classification model, of which the accuracy was 0.89 and the AUC was 0.93. The results of the evaluation metrics for machine learning are presented in Table S3.

## 4 Discussion

Based on the results of our FC analysis, network analysis, and graph theoretical analysis, we found that the self‐referential‐related brain regions of DPD participants showed significant alterations compared to those of HC participants.

In the whole‐brain FC analysis, the following FC results were consistent with those of the volume‐based and surface‐based analysis: between the left orbital part of IFG and left SFG; between the left orbital part of IFG and right MCC; between the right middle cingulate and the right MFG. However, the result between subcortical brain regions was only involved in the volume‐based analysis. Therefore, the results would be regarded as functionally important when the volume‐based FC analysis is consistent with the network analysis. Accordingly, the functionally important comparisons in the volume‐based FC were the left orbital part of IFG versus the left insula and the right MTG versus the left insula. In the network analysis, we regarded the increase in FC between the left supracallosal of ACC and the left orbital part of IFG as important due to its relatively large effect size. We also marked the importance of the insula due to the left insula having the largest node degree. Overall, the combined results of whole‐brain, network, and regional graph theoretical analysis suggest that the ACC, insula, and mPFC, which are implicated in self‐referential processing, may play a role in the altered self‐experience observed in DPD. However, additional research is necessary to clarify the specific contributions of these brain regions to the mechanisms of DPD, as similar regional alterations are also seen in other conditions, such as schizophrenia (Lee et al. 2016; Subramaniam 2021; Wylie and Tregellas 2010).

The performance of the KNN model indicated that the differential self‐referential‐related network and graph properties could be potential clinically applicable biomarkers for DPD diagnosis due to the relatively high accuracy of the model at 0.89 and the relatively large AUC at 0.93. In other words, if we diagnosed DPD using only differential self‐referential‐related network and graph properties, we could reach an accuracy of 0.89. This high accuracy was unexpected. This potential clinical application aligns with our earlier findings (Zheng et al. 2022), which indicate that disruptions in self‐referential networks and topological properties may contribute to the mechanisms underlying DPD. However, further studies involving control groups with altered self‐experiences or self‐disorders are essential to determine whether these brain regions are specifically associated with DPD.

Our results are in line with some previous evidence from the task‐related fMRI and PETs that highlight CMS abnormalities in DPD. The prior task‐related fMRI introduced above (Ketay et al. 2014) has demonstrated complex neural alterations associated with self‐processing in DPD. This study reveals distinctive patterns of neural activation, particularly in regions critical to self‐referential processing and emotional regulation. Moreover, studies have shown that the emotional detachment of DPD may be caused by abnormal decreases in limbic activity (hypothalamus and amygdala) and increased activity in BA 9 (DLPFC and dorsomedial prefrontal cortex) (Lemche et al. 2008, 2007, 2008, 2007).

The consistent observations of abnormal CMS activity in DPD point to the potential roles of these structures in the neural mechanisms of DPD. The CMS, including regions such as the ACC and mPFC, is central to processing self‐referential stimuli and generating a model of the self (Feng et al. 2018; Northoff and Bermpohl 2004; Weiler et al. 2016). In the present study, the key mPFC and ACC abnormalities compared to controls may be associated with alterations in evaluation, judgment, and monitoring in modeling the self. These may phenomenologically contribute to a range of abnormal experiences in DPD, such as feelings of unfamiliarity when confronted with self‐relevant materials (e.g., self‐name, self‐voice, self‐face), aberrant self‐observation, and a diminished sense of agency.

The present study further showed a negative correlation between the severity of the unreality of surroundings and the FC between the left IFG pars orbitalis and the left insula. The unreality of surroundings, aligning with the symptomatological connotations of derealization, implies a feeling of unreality and detachment from one's surroundings. According to the Semi‐Structured Clinical Interview for Dissociative Symptoms and Disorders (SCID‐D interview) (Steinberg 2023), such feelings include an experience of unfamiliarity with acquaintances or even having difficulties in recognizing them. In previous studies, both IFG and insula activity have been shown to be modulated by the strength of perceived familiarity with stimuli (Montaldi et al. 2006; Ranganath et al. 2004). Therefore, the diminished FC between IFG and insula may be involved in altered familiarity with previously familiar objects in patients with DPD, contributing to a sense of unreality in the environment. Furthermore, the recognition of objects involves not only the process of generating a sense of familiarity but also the emotional experience that accompanies the initial encoding of the object (Skinner and Fernandes 2007). A study by Jabbi and Keysers (2008) showed that IFG activity could have a causal effect on the anterior insula when one observes a facial expression, thus producing a corresponding emotional response in the subject. Therefore, reduced FC between the IFG and the insula may also indicate an abnormality in the generation of emotional responses in facing emotional stimuli. This may be related to the patient's detached, disconnected experience of the external environment, especially emotional scenes.

It was also shown that the FC between the left pregenual of the ACC and the left subgenual part of the ACC was negatively correlated with the severity of “perceptual alterations.” This includes sensory distortions such as feeling very light in the body, altered tactile perceptions, and a change in the size of the hands. It is recognized that the pregenual ACC and the subgenual ACC are implicated in visceral motor function and nociceptive processing and are negatively correlated with sensorimotor processing (Gradone et al. 2023; Stevens 2011; Vogt 2005; Yu et al. 2011). Thus, the changed FC between the ACC subregions could suggest an underlying neural mechanism that is involved in the altered experience of various sensory modalities in patients with DPD.

The mPFC, one of the key brain regions found in our study, on the other hand, is also shown to be involved in the default mode network. Since the default mode network has relatively higher activation in resting‐state or mind‐wandering, the mPFC was also described as “rest‐self overlap” (Bai et al. 2016). This suggested that mPFC plays an important role in both self‐referential processing and resting/mind‐wandering states. The abnormal functioning of mPFC might explain why DPD individuals describe that they were living in a dream, a state like mind‐wandering. Because these findings regarding the mPFC are aligned with the conclusion of our previous meta‐analysis (Zheng et al. 2022), where we identified mPFC as one of the key regions for noninvasive brain stimulation, the key role of mPFC in the mechanism of DPD is further confirmed.

Regarding external validity, our results can be generalized and provide empirical support for other similar theoretical models of DPD. For one thing, our results support the three‐level model (Qin et al. 2021), which proposed the link between bodily, environmental, and mental states in the self. This link is composed of three levels: interoceptive processing, which represents internal sensory signals (e.g., heartbeat); exteroceptive processing, which processing represents exteroceptive and proprioceptive signals (e.g., visual, auditory, and tactile); and mental self‐processing, which represents self‐related non‐bodily signals (e.g., personal traits). The model supposed that information about the bodily and external environment is integrated (exteroceptive processing) for the self via propagation from interoceptive processing to mental self‐processing. The three‐level model was one of the theories that explained the phenomenon of different self‐processing. Furthermore, they proposed that the insula is involved in any three‐level, the mPFC is involved in exteroceptive processing and mental self‐processing, and the ACC is involved in mental self‐processing. Abnormalities of interoception, exteroception, and mental self could be observed in patients with DPD. For instance, patients may describe experiencing visual distortions, such as things appearing flat, feeling that their bodies are unusually light, or finding that previously enjoyable activities no longer bring pleasure (Hunter et al. 2017; Sierra and Berrios 2000). Interoceptive processing, which represents internal sensory signals, has emerged as a crucial dimension in understanding DPD pathophysiology. Previous studies have systematically investigated alterations in interoceptive processing, revealing significant physiological disruptions. Previous studies (Giesbrecht et al. 2010; Michal et al. 2013; Monde et al. 2013) demonstrated that DPD patients exhibit profound autonomic nervous system dysregulation, characterized by abnormal skin conductance responses and reduced heart rate variability. These findings suggest a fundamental disturbance in bodily self‐perception at the interoceptive level. Our study found that ACC and mPFC could be key brain regions in the mechanisms of DPD, a disorder with self‐referential processing altered. Thus, our result could support the former theory.

On another level, our results support the use of a predictive coding framework to explain the mechanisms of DPD. Predictive coding posits that the brain generates predictions about sensory input based on prior experiences and beliefs, dynamically updating these predictions to minimize errors between expected and actual sensory input (Owens et al. 2018; Seth 2013). In the context of DPD, Gatus et al. (2022) propose that depersonalization and derealization symptoms could be manipulated individually by altering the relative precision of interoceptive predictions driven by exteroceptive, proprioceptive, and interoceptive sensory modalities. Their work highlights the roles of insula and ACC activation in integrating perception with physiological responses (Gatus et al. 2022). Gerrans (2019) applies the predictive coding framework to DPD, proposing that hypoactivity in the anterior insula cortex (AIC) disrupts the integration of predicted emotional responses with sensory and bodily inputs. This disruption results in a loss of affective meaning, leaving individuals feeling detached from their experiences. The key regions identified in our study—ACC, mPFC, and insula—are consistent with those implicated in these predictive coding hypotheses. Hence, our research provides neural evidence for the hypothesis.

Although these two hypotheses are different, they are not contradictory. By combining the structure of the three‐level model with the predictive coding explanation (Gatus et al. 2022; Gerrans 2019; Qin et al. 2021), we can potentially propose that in healthy people, the self‐reference of any given signal is formed by iteratively comparing the signals with the predicted self‐reference of that signal (through interoceptive‐ to mental‐self‐processing) and by dynamically updating the predictions to minimize prediction errors, resulting in both bottom‐up and top‐down modulations (Qin et al. 2021). In individuals with DPD, any level of self‐processing could be impaired and lead to altered interceptive prediction, which ultimately results in symptoms of depersonalization and derealization. To verify the hypotheses, we could use virtual reality and heartbeat‐evoked potential to test those suggested by Gatus et al. (2022) in the future.

There are several limitations to this study. First, our study focused on resting‐state fMRI, which limited our ability to explore the full effects of self‐referential tasks on DPD patients. We will explore it in the future using event‐related fMRI or other psychophysical methods. Second, we could not distinguish the different mechanisms of different symptoms of DPD. The correlation results of this study might just indicate that the state of the patients was related to the altered resting‐state fMRI features. Maybe they can be distinguished based on the method suggested by Gatus et al. (2022) in the future. Third, we did not control for potential confounding effects of disease duration and psychotropic medication for the limited sample size. Future research should systematically assess these variables and their potential impacts on FC, potentially stratifying the analysis by disease duration or medication status to disentangle their specific effects.

## 5 Conclusions

This is the first study to use resting‐state fMRI to identify alterations in self‐referential networks and topological properties in patients with DPD. Areas of the CMS involved in self‐referential processing (ACC and mPFC) and the insula were identified as important brain regions in the mechanisms of DPD. The high accuracy and AUC of the KNN model further indicate that these neural alterations could serve as potential biomarkers for DPD diagnosis. Our study also offers a neural basis for further research to verify the current three‐level model and predictive coding framework of DPD.

Clinically, the identification of abnormalities in ACC, mPFC, and insula, and the observed correlations between FC within self‐referential processing regions and DPD symptoms, offer new insights into the neurobiological underpinnings of DPD. This suggests that therapeutic strategies specifically targeting these regions and related networks could be promising. Interventions such as neuromodulation techniques or pharmacological treatments aimed at normalizing activity in these brain areas may hold potential in managing DPD symptoms.

## Author Contributions

**Yuan Jia**: conceptualization, validation, writing–original draft, writing–review and editing. **Nan Song**: validation, investigation, writing–review and editing. **Yanzhe Ning**: formal analysis, writing–review and editing. **Hong Zhu**: investigation, writing–review and editing, funding acquisition. **Linrui Dong**: data curation, writing–review and editing. **Sitong Feng**: data curation, writing–review and editing, visualization. **Hongxiao Jia**: conceptualization, resources, writing–review and editing. **Mingkang Song**: conceptualization, investigation, writing–review and editing. **Sisi Zheng**: conceptualization, methodology, software, validation, writing–review and editing, funding acquisition.

## Ethics Statement

The study was approved by the Ethical Committee of “Beijing Anding Hospital, Capital Medical University, China” No. 2020‐17.

## Consent

Informed consent was obtained from all subjects involved in the study.

## Conflicts of Interest

The authors declare no conflicts of interest.

### Peer Review

The peer review history for this article is available at https://publons.com/publon/10.1002/brb3.70314.

## Supporting information



Appendix A. Meta‐analysis results from Neurosynth
Appendix B. Meta‐analysis result
Appendix C. Graph theoretical analysis results

## Data Availability

The data presented in this study are available on request from the corresponding authors.
