# Supplementary material for: Altered Self‐Referential‐Related Brain Regions in Depersonalization‐Derealization Disorder
Source: Brain Behav. 2025 Feb 11;15(2):e70314. doi: 10.1002/brb3.70314 (PMC11813808; doi:10.1002/brb3.70314)
Supplement: Supplementary file 1 — Appendix A. Meta‐analysis results from Neurosynth Appendix B. Meta‐analysis result Appendix C. Graph theoretical analysis results [file BRB3-15-e70314-s001.docx]

**Appendix A. Meta-analysis results from Neurosynth**

In the terms of self-referential, 166 studies were included in the meta-analysis. Table S1 shows the title, authors and journal name of the papers.

**Table S1.** Studies included in the self-referential meta-analysis

| Title | Authors | Journal |
| --- | --- | --- |
| [Self-referential processing in unipolar depression: Distinct roles of subregions of the medial prefrontal cortex.](https://www.neurosynth.org/studies/28285207/) | Li Y, Li M, Wei D, Kong X, Du X, Hou X, Sun J, Qiu J | Psychiatry research. Neuroimaging |
| [Self-referential reflective activity and its relationship with rest: a PET study.](https://www.neurosynth.org/studies/15784441/) | D'Argembeau A, Collette F, Van der Linden M, Laureys S, Del Fiore G, Degueldre C, Luxen A, Salmon E | NeuroImage |
| [Default-mode network dysfunction and self-referential processing in healthy siblings of schizophrenia patients.](https://www.neurosynth.org/studies/23099059/) | van Buuren M, Vink M, Kahn RS | Schizophrenia research |
| [Rostral anterior cingulate cortex activity mediates the relationship between the depressive symptoms and the medial prefrontal cortex activity.](https://www.neurosynth.org/studies/19589603/) | Yoshimura S, Okamoto Y, Onoda K, Matsunaga M, Ueda K, Suzuki S, Shigetoyamawaki | Journal of affective disorders |
| [Directed forgetting of negative self-referential information is difficult: an FMRI study.](https://www.neurosynth.org/studies/24124475/) | Yang W, Liu P, Cui Q, Wei D, Li W, Qiu J, Zhang Q | PloS one |
| [Short-term escitalopram treatment normalizes aberrant self-referential processing in major depressive disorder.](https://www.neurosynth.org/studies/29747140/) | Komulainen E, Heikkila R, Nummenmaa L, Raij TT, Harmer CJ, Isometsa E, Ekelund J | Journal of affective disorders |
| [The influence of valence and decision difficulty on self-referential processing.](https://www.neurosynth.org/studies/23450237/) | Meffert H, Blanken L, Blair KS, White SF, Blair JR | Frontiers in human neuroscience |
| [Reduced functional coupling in the default-mode network during self-referential processing.](https://www.neurosynth.org/studies/20108218/) | van Buuren M, Gladwin TE, Zandbelt BB, Kahn RS, Vink M | Human brain mapping |
| [Decreased medial prefrontal cortex activation during self-referential processing in bipolar mania.](https://www.neurosynth.org/studies/28551555/) | Herold D, Usnich T, Spengler S, Sajonz B, Bauer M, Bermpohl F | Journal of affective disorders |
| [Delineating self-referential processing from episodic memory retrieval: common and dissociable networks.](https://www.neurosynth.org/studies/20123026/) | Sajonz B, Kahnt T, Margulies DS, Park SQ, Wittmann A, Stoy M, Strohle A, Heinz A, Northoff G, Bermpohl F | NeuroImage |
| [Antidepressant short-term and long-term brain effects during self-referential processing in major depression.](https://www.neurosynth.org/studies/26655583/) | Delaveau P, Jabourian M, Lemogne C, Allaili N, Choucha W, Girault N, Lehericy S, Laredo J, Fossati P | Psychiatry research. Neuroimaging |
| [A dual-subsystem model of the brain's default network: self-referential processing, memory retrieval processes, and autobiographical memory retrieval.](https://www.neurosynth.org/studies/22446489/) | Kim H | NeuroImage |
| [Reduced self-referential neural response during intergroup competition predicts competitor harm.](https://www.neurosynth.org/studies/24726338/) | Cikara M, Jenkins AC, Dufour N, Saxe R | NeuroImage |
| [Negative affectivity, self-referential processing and the cortical midline structures.](https://www.neurosynth.org/studies/20519253/) | Lemogne C, Gorwood P, Bergouignan L, Pelissolo A, Lehericy S, Fossati P | Social cognitive and affective neuroscience |
| [Self-referential and anxiety-relevant information processing in subclinical social anxiety: an fMRI study.](https://www.neurosynth.org/studies/22773051/) | Abraham A, Kaufmann C, Redlich R, Hermann A, Stark R, Stevens S, Hermann C | Brain imaging and behavior |
| [Functional specialization within the anterior medial prefrontal cortex: a functional magnetic resonance imaging study with human subjects.](https://www.neurosynth.org/studies/12531463/) | Zysset S, Huber O, Samson A, Ferstl EC, von Cramon DY | Neuroscience letters |
| [Self-referential processing of negative stimuli within the ventral anterior cingulate gyrus and right amygdala.](https://www.neurosynth.org/studies/18723260/) | Yoshimura S, Ueda K, Suzuki S, Onoda K, Okamoto Y, Yamawaki S | Brain and cognition |
| [The Queen and I: neural correlates of altered self-related cognitions in major depressive episode.](https://www.neurosynth.org/studies/24205330/) | Sarsam M, Parkes LM, Roberts N, Reid GS, Kinderman P | PloS one |
| [Neural activity during self-referential working memory and the underlying role of the amygdala in social anxiety disorder.](https://www.neurosynth.org/studies/27260987/) | Yoon HJ, Kim JS, Shin YB, Choi SH, Lee SK, Kim JJ | Neuroscience letters |
| [Imagining triadic interactions simultaneously activates mirror and mentalizing systems.](https://www.neurosynth.org/studies/24825504/) | Trapp K, Spengler S, Wustenberg T, Wiers CE, Busch NA, Bermpohl F | NeuroImage |
| [Medial cortex activity, self-reflection and depression.](https://www.neurosynth.org/studies/19620180/) | Johnson MK, Nolen-Hoeksema S, Mitchell KJ, Levin Y | Social cognitive and affective neuroscience |
| [Plastic modulation of episodic memory networks in the aging brain with cognitive decline.](https://www.neurosynth.org/studies/27091676/) | Bai F, Yuan Y, Yu H, Zhang Z | Behavioural brain research |
| [Medial prefrontal cortex and self-referential mental activity: relation to a default mode of brain function.](https://www.neurosynth.org/studies/11259662/) | Gusnard DA, Akbudak E, Shulman GL, Raichle ME | Proceedings of the National Academy of Sciences of the United States of America |
| [Self-referential processing influences functional activation during cognitive control: an fMRI study.](https://www.neurosynth.org/studies/22798398/) | Wagner G, Koch K, Schachtzabel C, Peikert G, Schultz CC, Reichenbach JR, Sauer H, Schlosser RG | Social cognitive and affective neuroscience |
| [Self, mother and abstract other: an fMRI study of reflective social processing.](https://www.neurosynth.org/studies/18486489/) | Vanderwal T, Hunyadi E, Grupe DW, Connors CM, Schultz RT | NeuroImage |
| [Self-referential processing and the prefrontal cortex over the course of depression: a pilot study.](https://www.neurosynth.org/studies/19945172/) | Lemogne C, Mayberg H, Bergouignan L, Volle E, Delaveau P, Lehericy S, Allilaire JF, Fossati P | Journal of affective disorders |
| [Guilt-specific processing in the prefrontal cortex.](https://www.neurosynth.org/studies/21427167/) | Wagner U, N'Diaye K, Ethofer T, Vuilleumier P | Cerebral cortex (New York, N.Y. : 1991) |
| [In search of the depressive self: extended medial prefrontal network during self-referential processing in major depression.](https://www.neurosynth.org/studies/19307251/) | Lemogne C, le Bastard G, Mayberg H, Volle E, Bergouignan L, Lehericy S, Allilaire JF, Fossati P | Social cognitive and affective neuroscience |
| [The neural basis of the abnormal self-referential processing and its impact on cognitive control in depressed patients.](https://www.neurosynth.org/studies/25872899/) | Wagner G, Schachtzabel C, Peikert G, Bar KJ | Human brain mapping |
| [Neuroimaging self-esteem: a fMRI study of individual differences in women.](https://www.neurosynth.org/studies/22403154/) | Frewen PA, Lundberg E, Brimson-Theberge M, Theberge J | Social cognitive and affective neuroscience |
| [Neural substrates of self-referential processing in Chinese Buddhists.](https://www.neurosynth.org/studies/19620181/) | Han S, Gu X, Mao L, Ge J, Wang G, Ma Y | Social cognitive and affective neuroscience |
| [Neural correlates of self-referential processing and implicit self-associations in chronic depression.](https://www.neurosynth.org/studies/26226432/) | Renner F, Siep N, Lobbestael J, Arntz A, Peeters FP, Huibers MJ | Journal of affective disorders |
| [Reduced neural differentiation between self-referential cognitive and emotional processes in women with borderline personality disorder.](https://www.neurosynth.org/studies/26231122/) | Scherpiet S, Herwig U, Opialla S, Scheerer H, Habermeyer V, Jancke L, Bruhl AB | Psychiatry research |
| [Altered resting state connectivity of the default mode network in alexithymia.](https://www.neurosynth.org/studies/22563009/) | Liemburg EJ, Swart M, Bruggeman R, Kortekaas R, Knegtering H, Curcic-Blake B, Aleman A | Social cognitive and affective neuroscience |
| [Large-scale directional connections among multi resting-state neural networks in human brain: a functional MRI and Bayesian network modeling study.](https://www.neurosynth.org/studies/21396456/) | Li R, Chen K, Fleisher AS, Reiman EM, Yao L, Wu X | NeuroImage |
| [Neural representation and clinically relevant moderators of individualised self-criticism in healthy subjects.](https://www.neurosynth.org/studies/23887820/) | Doerig N, Schlumpf Y, Spinelli S, Spati J, Brakowski J, Quednow BB, Seifritz E, Grosse Holtforth M | Social cognitive and affective neuroscience |
| [Atypical modulation of medial prefrontal cortex to self-referential comments in generalized social phobia.](https://www.neurosynth.org/studies/21601433/) | Blair KS, Geraci M, Otero M, Majestic C, Odenheimer S, Jacobs M, Blair RJ, Pine DS | Psychiatry research |
| [Brain activity and functional coupling changes associated with self-reference effect during both encoding and retrieval.](https://www.neurosynth.org/studies/24608131/) | Morel N, Villain N, Rauchs G, Gaubert M, Piolino P, Landeau B, Mezenge F, Desgranges B, Eustache F, Chetelat G | PloS one |
| [Neural networks involved in self-judgement in young and elderly adults.](https://www.neurosynth.org/studies/20594938/) | Feyers D, Collette F, D'Argembeau A, Majerus S, Salmon E | NeuroImage |
| [Bipolar and borderline patients display differential patterns of functional connectivity among resting state networks.](https://www.neurosynth.org/studies/24793833/) | Das P, Calhoun V, Malhi GS | NeuroImage |
| [Impact of meditation training on the default mode network during a restful state.](https://www.neurosynth.org/studies/22446298/) | Taylor VA, Daneault V, Grant J, Scavone G, Breton E, Roffe-Vidal S, Courtemanche J, Lavarenne AS, Marrelec G, Benali H, Beauregard M | Social cognitive and affective neuroscience |
| [Cognitive appraisal and life stress moderate the effects of the 5-HTTLPR polymorphism on amygdala reactivity.](https://www.neurosynth.org/studies/21246665/) | Lemogne C, Gorwood P, Boni C, Pessiglione M, Lehericy S, Fossati P | Human brain mapping |
| [Distributed self in episodic memory: neural correlates of successful retrieval of self-encoded positive and negative personality traits.](https://www.neurosynth.org/studies/15275916/) | Fossati P, Hevenor SJ, Lepage M, Graham SJ, Grady C, Keightley ML, Craik F, Mayberg H | NeuroImage |
| [Reflections of Oneself: Neurocognitive Evidence for Dissociable Forms of Self-Referential Recollection.](https://www.neurosynth.org/studies/24700584/) | Bergstrom ZM, Vogelsang DA, Benoit RG, Simons JS | Cerebral cortex (New York, N.Y. : 1991) |
| [Impact of Mindfulness-Based Stress Reduction training on intrinsic brain connectivity.](https://www.neurosynth.org/studies/21334442/) | Kilpatrick LA, Suyenobu BY, Smith SR, Bueller JA, Goodman T, Creswell JD, Tillisch K, Mayer EA, Naliboff BD | NeuroImage |
| [Self-related awareness and emotion regulation.](https://www.neurosynth.org/studies/20045475/) | Herwig U, Kaffenberger T, Jancke L, Bruhl AB | NeuroImage |
| [Structural but not functional neuroplasticity one year after effective cognitive behaviour therapy for social anxiety disorder.](https://www.neurosynth.org/studies/27838341/) | Mansson KNT, Salami A, Carlbring P, Boraxbekk CJ, Andersson G, Furmark T | Behavioural brain research |
| [Childhood emotional maltreatment severity is associated with dorsal medial prefrontal cortex responsivity to social exclusion in young adults.](https://www.neurosynth.org/studies/24416347/) | van Harmelen AL, Hauber K, Gunther Moor B, Spinhoven P, Boon AE, Crone EA, Elzinga BM | PloS one |
| [Default mode network dissociation in depressive and anxiety states.](https://www.neurosynth.org/studies/25804311/) | Coutinho JF, Fernandesl SV, Soares JM, Maia L, Goncalves OF, Sampaio A | Brain imaging and behavior |
| [Escitalopram attenuates posterior cingulate activity during self-evaluation in healthy volunteers.](https://www.neurosynth.org/studies/20418072/) | Matthews SC, Simmons AN, Strigo IA, Arce E, Stein MB, Paulus MP | Psychiatry research |
| [Resting-state synchrony between anterior cingulate cortex and precuneus relates to body shape concern in anorexia nervosa and bulimia nervosa.](https://www.neurosynth.org/studies/24300085/) | Lee S, Ran Kim K, Ku J, Lee JH, Namkoong K, Jung YC | Psychiatry research |
| [Atypical neural self-representation in autism.](https://www.neurosynth.org/studies/20008375/) | Lombardo MV, Chakrabarti B, Bullmore ET, Sadek SA, Pasco G, Wheelwright SJ, Suckling J, Baron-Cohen S | Brain : a journal of neurology |
| [Contagious yawning and the brain.](https://www.neurosynth.org/studies/15820652/) | Platek SM, Mohamed FB, Gallup GG Jr | Brain research. Cognitive brain research |
| [Oxytocin effects on self-referential processing: behavioral and neuroimaging evidence.](https://www.neurosynth.org/studies/29040763/) | Liu Y, Wu B, Wang X, Li W, Zhang T, Wu X, Han S | Social cognitive and affective neuroscience |
| [Neurobiology of self-awareness in schizophrenia: an fMRI study.](https://www.neurosynth.org/studies/22480958/) | Shad MU, Keshavan MS, Steinberg JL, Mihalakos P, Thomas BP, Motes MA, Soares JC, Tamminga CA | Schizophrenia research |
| [Imbalance of default mode and regulatory networks during externally focused processing in depression.](https://www.neurosynth.org/studies/25274576/) | Belleau EL, Taubitz LE, Larson CL | Social cognitive and affective neuroscience |
| [Self processing in the brain: A paradigmatic fMRI case study with a professional singer.](https://www.neurosynth.org/studies/24732954/) | Zaytseva Y, Gutyrchik E, Bao Y, Poppel E, Han S, Northoff G, Welker L, Meindl T, Blautzik J | Brain and cognition |
| [Episodic memory and self-reference via semantic autobiographical memory: insights from an fMRI study in younger and older adults.](https://www.neurosynth.org/studies/25628546/) | Kalenzaga S, Sperduti M, Anssens A, Martinelli P, Devauchelle AD, Gallarda T, Delhommeau M, Lion S, Amado I, Krebs MO, Oppenheim C, Piolino P | Frontiers in behavioral neuroscience |
| [Dissociable medial prefrontal contributions to judgments of similar and dissimilar others.](https://www.neurosynth.org/studies/16701214/) | Mitchell JP, Macrae CN, Banaji MR | Neuron |
| [Neuroticism modulates amygdala-prefrontal connectivity in response to negative emotional facial expressions.](https://www.neurosynth.org/studies/19683585/) | Cremers HR, Demenescu LR, Aleman A, Renken R, van Tol MJ, van der Wee NJ, Veltman DJ, Roelofs K | NeuroImage |
| [Neuroimaging social emotional processing in women: fMRI study of script-driven imagery.](https://www.neurosynth.org/studies/20525743/) | Frewen PA, Dozois DJ, Neufeld RW, Densmore M, Stevens TK, Lanius RA | Social cognitive and affective neuroscience |
| [Are autobiographical memories inherently social? Evidence from an fMRI study.](https://www.neurosynth.org/studies/23028774/) | Wilbers L, Deuker L, Fell J, Axmacher N | PloS one |
| [The neural sociometer: brain mechanisms underlying state self-esteem.](https://www.neurosynth.org/studies/21452934/) | Eisenberger NI, Inagaki TK, Muscatell KA, Byrne Haltom KE, Leary MR | Journal of cognitive neuroscience |
| [Functional brain networks involved in reality monitoring.](https://www.neurosynth.org/studies/26004062/) | Metzak PD, Lavigne KM, Woodward TS | Neuropsychologia |
| [General and emotion-specific alterations to cognitive control in women with a history of childhood abuse.](https://www.neurosynth.org/studies/28794976/) | Mackiewicz Seghete KL, Kaiser RH, DePrince AP, Banich MT | NeuroImage. Clinical |
| [The Self-Pleasantness Judgment Modulates the Encoding Performance and the Default Mode Network Activity.](https://www.neurosynth.org/studies/27047364/) | Perrone-Bertolotti M, Cerles M, Ramdeen KT, Boudiaf N, Pichat C, Hot P, Baciu M | Frontiers in human neuroscience |
| [The neural correlates of reciprocity are sensitive to prior experience of reciprocity.](https://www.neurosynth.org/studies/28551067/) | Caceda R, Prendes-Alvarez S, Hsu JJ, Tripathi SP, Kilts CD, James GA | Behavioural brain research |
| [A Coordinate-Based Meta-Analysis of Overlaps in Regional Specialization and Functional Connectivity across Subjective Value and Default Mode Networks.](https://www.neurosynth.org/studies/28154520/) | Acikalin MY, Gorgolewski KJ, Poldrack RA | Frontiers in neuroscience |
| [Increased Visual Stimulation Systematically Decreases Activity in Lateral Intermediate Cortex.](https://www.neurosynth.org/studies/25480358/) | Nasr S, Stemmann H, Vanduffel W, Tootell RB | Cerebral cortex (New York, N.Y. : 1991) |
| [Altered resting state networks in epileptic patients with generalized tonic-clonic seizures.](https://www.neurosynth.org/studies/21167825/) | Wang Z, Lu G, Zhang Z, Zhong Y, Jiao Q, Zhang Z, Tan Q, Tian L, Chen G, Liao W, Li K, Liu Y | Brain research |
| [Neural correlates of pragmatic language comprehension in autism spectrum disorders.](https://www.neurosynth.org/studies/19423680/) | Tesink CM, Buitelaar JK, Petersson KM, van der Gaag RJ, Kan CC, Tendolkar I, Hagoort P | Brain : a journal of neurology |
| [Racial identification modulates default network activity for same and other races.](https://www.neurosynth.org/studies/21618667/) | Mathur VA, Harada T, Chiao JY | Human brain mapping |
| [Brain activation patterns during memory of cognitive agency.](https://www.neurosynth.org/studies/16516497/) | Vinogradov S, Luks TL, Simpson GV, Schulman BJ, Glenn S, Wong AE | NeuroImage |
| [Neural correlates of mindful self-awareness in mindfulness meditators and meditation-naive subjects revisited.](https://www.neurosynth.org/studies/27377788/) | Lutz J, Bruhl AB, Scheerer H, Jancke L, Herwig U | Biological psychology |
| [Neural processes underlying memory attribution on a reality-monitoring task.](https://www.neurosynth.org/studies/16648457/) | Kensinger EA, Schacter DL | Cerebral cortex (New York, N.Y. : 1991) |
| [Neural substrates of interpreting actions and emotions from body postures.](https://www.neurosynth.org/studies/21504992/) | Kana RK, Travers BG | Social cognitive and affective neuroscience |
| [Equivalent neural responses in children and adolescents with and without autism during judgments of affect.](https://www.neurosynth.org/studies/24016745/) | Vander Wyk BC, Hoffman F, Pelphrey KA | Developmental cognitive neuroscience |
| [Task-positive and task-negative networks and their relation to depression: EEG beamformer analysis.](https://www.neurosynth.org/studies/27001453/) | Knyazev GG, Savostyanov AN, Bocharov AV, Tamozhnikov SS, Saprigyn AE | Behavioural brain research |
| [Prefrontal transcranial direct current stimulation changes connectivity of resting-state networks during fMRI.](https://www.neurosynth.org/studies/22031874/) | Keeser D, Meindl T, Bor J, Palm U, Pogarell O, Mulert C, Brunelin J, Moller HJ, Reiser M, Padberg F | The Journal of neuroscience : the official journal of the Society for Neuroscience |
| [Oxytocin facilitates the sensation of social stress.](https://www.neurosynth.org/studies/24659430/) | Eckstein M, Scheele D, Weber K, Stoffel-Wagner B, Maier W, Hurlemann R | Human brain mapping |
| [Neural response to eye contact and paroxetine treatment in generalized social anxiety disorder.](https://www.neurosynth.org/studies/22047726/) | Schneier FR, Pomplun M, Sy M, Hirsch J | Psychiatry research |
| [Dispositional mindfulness is predicted by structural development of the insula during late adolescence.](https://www.neurosynth.org/studies/26209810/) | Friedel S, Whittle SL, Vijayakumar N, Simmons JG, Byrne ML, Schwartz OS, Allen NB | Developmental cognitive neuroscience |
| [Neural correlates of autobiographical problem-solving deficits associated with rumination in depression.](https://www.neurosynth.org/studies/28477499/) | Jones NP, Fournier JC, Stone LB | Journal of affective disorders |
| [The maturing architecture of the brain's default network.](https://www.neurosynth.org/studies/18322013/) | Fair DA, Cohen AL, Dosenbach NU, Church JA, Miezin FM, Barch DM, Raichle ME, Petersen SE, Schlaggar BL | Proceedings of the National Academy of Sciences of the United States of America |
| [Coping with emotions past: the neural bases of regulating affect associated with negative autobiographical memories.](https://www.neurosynth.org/studies/19058792/) | Kross E, Davidson M, Weber J, Ochsner K | Biological psychiatry |
| [Hippocampal contributions to the processing of social emotions.](https://www.neurosynth.org/studies/22012639/) | Immordino-Yang MH, Singh V | Human brain mapping |
| [Neural correlates of experimentally induced flow experiences.](https://www.neurosynth.org/studies/23959200/) | Ulrich M, Keller J, Hoenig K, Waller C, Gron G | NeuroImage |
| [Spontaneous low-frequency BOLD signal fluctuations: an fMRI investigation of the resting-state default mode of brain function hypothesis.](https://www.neurosynth.org/studies/15852468/) | Fransson P | Human brain mapping |
| [Mindfulness practice leads to increases in regional brain gray matter density.](https://www.neurosynth.org/studies/21071182/) | Holzel BK, Carmody J, Vangel M, Congleton C, Yerramsetti SM, Gard T, Lazar SW | Psychiatry research |
| [Psychological and neural mechanisms of subjective time dilation.](https://www.neurosynth.org/studies/21559346/) | van Wassenhove V, Wittmann M, Craig AD, Paulus MP | Frontiers in neuroscience |
| [The neural basis for understanding non-intended actions.](https://www.neurosynth.org/studies/17499159/) | Buccino G, Baumgaertner A, Colle L, Buechel C, Rizzolatti G, Binkofski F | NeuroImage |
| [Neural correlates of moral reasoning in autism spectrum disorder.](https://www.neurosynth.org/studies/22569187/) | Schneider K, Pauly KD, Gossen A, Mevissen L, Michel TM, Gur RC, Schneider F, Habel U | Social cognitive and affective neuroscience |
| [Patterns of brain activity supporting autobiographical memory, prospection, and theory of mind, and their relationship to the default mode network.](https://www.neurosynth.org/studies/19580387/) | Spreng RN, Grady CL | Journal of cognitive neuroscience |
| [Traumatic brain injury affects the frontomedian cortex--an event-related fMRI study on evaluative judgments.](https://www.neurosynth.org/studies/19747929/) | Schroeter ML, Ettrich B, Menz M, Zysset S | Neuropsychologia |
| [Neural substrates of cognitive control under the belief of getting neurofeedback training.](https://www.neurosynth.org/studies/24421765/) | Ninaus M, Kober SE, Witte M, Koschutnig K, Stangl M, Neuper C, Wood G | Frontiers in human neuroscience |
| [Gender differences in creative thinking: behavioral and fMRI findings.](https://www.neurosynth.org/studies/23807175/) | Abraham A, Thybusch K, Pieritz K, Hermann C | Brain imaging and behavior |
| [Task-positive and task-negative networks in major depressive disorder: A combined fMRI and EEG study.](https://www.neurosynth.org/studies/29656269/) | Knyazev GG, Savostyanov AN, Bocharov AV, Brak IV, Osipov EA, Filimonova EA, Saprigyn AE, Aftanas LI | Journal of affective disorders |
| [Adolescent Gender Differences in Cognitive Control Performance and Functional Connectivity Between Default Mode and Fronto-Parietal Networks Within a Self-Referential Context.](https://www.neurosynth.org/studies/29740292/) | Alarcon G, Pfeifer JH, Fair DA, Nagel BJ | Frontiers in behavioral neuroscience |
| [Aberrant functional connectivity of resting state networks in transient ischemic attack.](https://www.neurosynth.org/studies/23951069/) | Li R, Wang S, Zhu L, Guo J, Zeng L, Gong Q, He L, Chen H | PloS one |
| [Moral decision-making, ToM, empathy and the default mode network.](https://www.neurosynth.org/studies/22459338/) | Reniers RL, Corcoran R, Vollm BA, Mashru A, Howard R, Liddle PF | Biological psychology |
| [Extended self: spontaneous activation of medial prefrontal cortex by objects that are 'mine'](https://www.neurosynth.org/studies/23696692/) | Kim K, Johnson MK | Social cognitive and affective neuroscience |
| [Alterations in amplitude of low frequency fluctuation in treatment-naive major depressive disorder measured with resting-state fMRI.](https://www.neurosynth.org/studies/24740815/) | Liu J, Ren L, Womer FY, Wang J, Fan G, Jiang W, Blumberg HP, Tang Y, Xu K, Wang F | Human brain mapping |
| [Long-term use of psychedelic drugs is associated with differences in brain structure and personality in humans.](https://www.neurosynth.org/studies/25637267/) | Bouso JC, Palhano-Fontes F, Rodriguez-Fornells A, Ribeiro S, Sanches R, Crippa JA, Hallak JE, de Araujo DB, Riba J | European neuropsychopharmacology : the journal of the European College of Neuropsychopharmacology |
| [Neural Processing of Familiar and Unfamiliar Children's Faces: Effects of Experienced Love Withdrawal, but No Effects of Neutral and Threatening Priming.](https://www.neurosynth.org/studies/27303279/) | Heckendorf E, Huffmeijer R, Bakermans-Kranenburg MJ, van IJzendoorn MH | Frontiers in human neuroscience |
| [Time is nothing: emotional consistency of autobiographical memory and its neural basis.](https://www.neurosynth.org/studies/28980133/) | Xu R, Yang J, Feng C, Wu H, Huang R, Yang Q, Li Z, Xu P, Gu R, Luo YJ | Brain imaging and behavior |
| [Altered function and connectivity of the medial frontal cortex in pediatric obsessive-compulsive disorder.](https://www.neurosynth.org/studies/20947065/) | Fitzgerald KD, Stern ER, Angstadt M, Nicholson-Muth KC, Maynor MR, Welsh RC, Hanna GL, Taylor SF | Biological psychiatry |
| [Deviant functional activation and connectivity of the right insula are associated with lack of awareness of episodic memory impairment in nonamnesic alcoholism.](https://www.neurosynth.org/studies/28806707/) | Le Berre AP, Muller-Oehring EM, Schulte T, Serventi MR, Pfefferbaum A, Sullivan EV | Cortex; a journal devoted to the study of the nervous system and behavior |
| [A relation between rest and the self in the brain?](https://www.neurosynth.org/studies/14572916/) | Wicker B, Ruby P, Royet JP, Fonlupt P | Brain research. Brain research reviews |
| [Emotion processing in the aging brain is modulated by semantic elaboration.](https://www.neurosynth.org/studies/20869375/) | Ritchey M, Bessette-Symons B, Hayes SM, Cabeza R | Neuropsychologia |
| [Semantic memory involvement in the default mode network: a functional neuroimaging study using independent component analysis.](https://www.neurosynth.org/studies/20965253/) | Wirth M, Jann K, Dierks T, Federspiel A, Wiest R, Horn H | NeuroImage |
| [Selective impairments of resting-state networks in minimal hepatic encephalopathy.](https://www.neurosynth.org/studies/22662152/) | Qi R, Zhang LJ, Xu Q, Zhong J, Wu S, Zhang Z, Liao W, Ni L, Zhang Z, Chen H, Zhong Y, Jiao Q, Wu X, Fan X, Liu Y, Lu G | PloS one |
| [Dissociable neural substrates for agentic versus conceptual representations of self.](https://www.neurosynth.org/studies/19925182/) | Powell LJ, Macrae CN, Cloutier J, Metcalfe J, Mitchell JP | Journal of cognitive neuroscience |
| [Dissociable neural systems supporting knowledge about human character and appearance in ourselves and others.](https://www.neurosynth.org/studies/20946059/) | Moran JM, Lee SM, Gabrieli JD | Journal of cognitive neuroscience |
| [Dissociable roles of default-mode regions during episodic encoding.](https://www.neurosynth.org/studies/24315838/) | Maillet D, Rajah MN | NeuroImage |
| [Dysregulation between emotion and theory of mind networks in borderline personality disorder.](https://www.neurosynth.org/studies/25482858/) | O'Neill A, D'Souza A, Samson AC, Carballedo A, Kerskens C, Frodl T | Psychiatry research |
| [Higher or lower? The functional anatomy of perceived allocentric social hierarchies.](https://www.neurosynth.org/studies/21664277/) | Farrow TF, Jones SC, Kaylor-Hughes CJ, Wilkinson ID, Woodruff PW, Hunter MD, Spence SA | NeuroImage |
| [Right supramarginal gyrus is crucial to overcome emotional egocentricity bias in social judgments.](https://www.neurosynth.org/studies/24068815/) | Silani G, Lamm C, Ruff CC, Singer T | The Journal of neuroscience : the official journal of the Society for Neuroscience |
| [Neural correlates of self-evaluative accuracy after traumatic brain injury.](https://www.neurosynth.org/studies/16154166/) | Schmitz TW, Rowley HA, Kawahara TN, Johnson SC | Neuropsychologia |
| [Processing of autobiographical memory retrieval cues in borderline personality disorder.](https://www.neurosynth.org/studies/16837057/) | Schnell K, Dietrich T, Schnitker R, Daumann J, Herpertz SC | Journal of affective disorders |
| [The neural architecture of music-evoked autobiographical memories.](https://www.neurosynth.org/studies/19240137/) | Janata P | Cerebral cortex (New York, N.Y. : 1991) |
| [Dreaming as mind wandering: evidence from functional neuroimaging and first-person content reports.](https://www.neurosynth.org/studies/23908622/) | Fox KC, Nijeboer S, Solomonova E, Domhoff GW, Christoff K | Frontiers in human neuroscience |
| [Functional connectivity in incarcerated male adolescents with psychopathic traits.](https://www.neurosynth.org/studies/28521281/) | Thijssen S, Kiehl KA | Psychiatry research. Neuroimaging |
| [Reduced amygdala reactivity and impaired working memory during dissociation in borderline personality disorder.](https://www.neurosynth.org/studies/28526931/) | Krause-Utz A, Winter D, Schriner F, Chiu CD, Lis S, Spinhoven P, Bohus M, Schmahl C, Elzinga BM | European archives of psychiatry and clinical neuroscience |
| [Nicotine effects on default mode network during resting state.](https://www.neurosynth.org/studies/21331518/) | Tanabe J, Nyberg E, Martin LF, Martin J, Cordes D, Kronberg E, Tregellas JR | Psychopharmacology |
| [Developmental changes in within- and between-network connectivity between late childhood and adulthood.](https://www.neurosynth.org/studies/23174403/) | Barber AD, Caffo BS, Pekar JJ, Mostofsky SH | Neuropsychologia |
| [From Vivaldi to Beatles and back: predicting lateralized brain responses to music.](https://www.neurosynth.org/studies/23810975/) | Alluri V, Toiviainen P, Lund TE, Wallentin M, Vuust P, Nandi AK, Ristaniemi T, Brattico E | NeuroImage |
| [Brain Correlates of Self-Evaluation Deficits in Schizophrenia: A Combined Functional and Structural MRI Study.](https://www.neurosynth.org/studies/26406464/) | Tan S, Zhao Y, Fan F, Zou Y, Jin Z, Zen Y, Zhu X, Yang F, Tan Y, Zhou D | PloS one |
| [Aging Affects the Interaction between Attentional Control and Source Memory: An fMRI Study.](https://www.neurosynth.org/studies/24800631/) | Dulas MR, Duarte A | Journal of cognitive neuroscience |
| [Neural correlates of emotional working memory in patients with mild cognitive impairment.](https://www.neurosynth.org/studies/17915264/) | Dohnel K, Sommer M, Ibach B, Rothmayr C, Meinhardt J, Hajak G | Neuropsychologia |
| [The neural basis of personal goal processing when envisioning future events.](https://www.neurosynth.org/studies/19642887/) | D'Argembeau A, Stawarczyk D, Majerus S, Collette F, Van der Linden M, Feyers D, Maquet P, Salmon E | Journal of cognitive neuroscience |
| [Encoding social interactions: the neural correlates of true and false memories.](https://www.neurosynth.org/studies/20433241/) | Straube B, Green A, Chatterjee A, Kircher T | Journal of cognitive neuroscience |
| [Thinking about the future versus the past in personal and non-personal contexts.](https://www.neurosynth.org/studies/18703030/) | Abraham A, Schubotz RI, von Cramon DY | Brain research |
| [When I think about me and simulate you: medial rostral prefrontal cortex and self-referential processes.](https://www.neurosynth.org/studies/20045478/) | Benoit RG, Gilbert SJ, Volle E, Burgess PW | NeuroImage |
| [Dissociating the roles of the default-mode, dorsal, and ventral networks in episodic memory retrieval.](https://www.neurosynth.org/studies/20097295/) | Kim H | NeuroImage |
| [Individualized and clinically derived stimuli activate limbic structures in depression: an fMRI study.](https://www.neurosynth.org/studies/21283580/) | Kessler H, Taubner S, Buchheim A, Munte TF, Stasch M, Kachele H, Roth G, Heinecke A, Erhard P, Cierpka M, Wiswede D | PloS one |
| [Adolescent resting state networks and their associations with schizotypal trait expression.](https://www.neurosynth.org/studies/20844603/) | Lagioia A, Van De Ville D, Debbane M, Lazeyras F, Eliez S | Frontiers in systems neuroscience |
| [Dynamic neural networks supporting memory retrieval.](https://www.neurosynth.org/studies/21550407/) | St Jacques PL, Kragel PA, Rubin DC | NeuroImage |
| [Neurocognitive processes of the religious leader in Christians.](https://www.neurosynth.org/studies/19507157/) | Ge J, Gu X, Ji M, Han S | Human brain mapping |
| [Pain enhances functional connectivity of a brain network evoked by performance of a cognitive task.](https://www.neurosynth.org/studies/17314240/) | Seminowicz DA, Davis KD | Journal of neurophysiology |
| [Neural correlates of self-perceptions in adolescents with major depressive disorder.](https://www.neurosynth.org/studies/26943454/) | Bradley KA, Colcombe S, Henderson SE, Alonso CM, Milham MP, Gabbay V | Developmental cognitive neuroscience |
| [Brain activation to task-irrelevant disorder-related threat in social anxiety disorder: The impact of symptom severity.](https://www.neurosynth.org/studies/28224080/) | Heitmann CY, Feldker K, Neumeister P, Brinkmann L, Schrammen E, Zwitserlood P, Straube T | NeuroImage. Clinical |
| [Neural basis of moral elevation demonstrated through inter-subject synchronization of cortical activity during free-viewing.](https://www.neurosynth.org/studies/22745745/) | Englander ZA, Haidt J, Morris JP | PloS one |
| [Altered resting brain connectivity in persistent cancer related fatigue.](https://www.neurosynth.org/studies/26106555/) | Hampson JP, Zick SM, Khabir T, Wright BD, Harris RE | NeuroImage. Clinical |
| [Self-construal differences in neural responses to negative social cues.](https://www.neurosynth.org/studies/28782584/) | Liddell BJ, Felmingham KL, Das P, Whitford TJ, Malhi GS, Battaglini E, Bryant RA | Biological psychology |
| [Remembering the past and imagining the future: common and distinct neural substrates during event construction and elaboration.](https://www.neurosynth.org/studies/17126370/) | Addis DR, Wong AT, Schacter DL | Neuropsychologia |
| [Differential role of the Mentalizing and the Mirror Neuron system in the imitation of communicative gestures.](https://www.neurosynth.org/studies/23684882/) | Mainieri AG, Heim S, Straube B, Binkofski F, Kircher T | NeuroImage |
| [The angular gyrus is a supramodal comparator area in action-outcome monitoring.](https://www.neurosynth.org/studies/28439662/) | van Kemenade BM, Arikan BE, Kircher T, Straube B | Brain structure & function |
| [Individual differences in risk preference predict neural responses during financial decision-making.](https://www.neurosynth.org/studies/19576868/) | Engelmann JB, Tamir D | Brain research |
| [Look who's judging-Feedback source modulates brain activation to performance feedback in social anxiety.](https://www.neurosynth.org/studies/27033687/) | Peterburs J, Sandrock C, Miltner WHR, Straube T | NeuroImage |
| [Intrinsic network connectivity and own body perception in gender dysphoria.](https://www.neurosynth.org/studies/27444730/) | Feusner JD, Lidstrom A, Moody TD, Dhejne C, Bookheimer SY, Savic I | Brain imaging and behavior |
| [How default is the default mode of brain function? Further evidence from intrinsic BOLD signal fluctuations.](https://www.neurosynth.org/studies/16879844/) | Fransson P | Neuropsychologia |
| [Top-down regulation of default mode activity in spatial visual attention.](https://www.neurosynth.org/studies/23575842/) | Wen X, Liu Y, Yao L, Ding M | The Journal of neuroscience : the official journal of the Society for Neuroscience |
| [Association of poor insight in schizophrenia with structure and function of cortical midline structures and frontopolar cortex.](https://www.neurosynth.org/studies/22664168/) | Raij TT, Riekki TJ, Hari R | Schizophrenia research |
| [Neural correlates of social cognition in naturalistic settings: a model-free analysis approach.](https://www.neurosynth.org/studies/19733672/) | Wolf I, Dziobek I, Heekeren HR | NeuroImage |
| [Medial prefrontal cortex supports source memory for self-referenced materials in young and older adults.](https://www.neurosynth.org/studies/23904335/) | Leshikar ED, Duarte A | Cognitive, affective & behavioral neuroscience |
| [Roles of medial prefrontal cortex and orbitofrontal cortex in self-evaluation.](https://www.neurosynth.org/studies/19925187/) | Beer JS, Lombardo MV, Bhanji JP | Journal of cognitive neuroscience |
| [Self-face evaluation and self-esteem in young females: an fMRI study using contrast effect.](https://www.neurosynth.org/studies/22079451/) | Oikawa H, Sugiura M, Sekiguchi A, Tsukiura T, Miyauchi CM, Hashimoto T, Takano-Yamamoto T, Kawashima R | NeuroImage |
| [Neural networks underlying language and social cognition during self-other processing in Autism spectrum disorders.](https://www.neurosynth.org/studies/28619530/) | Kana RK, Sartin EB, Stevens C Jr, Deshpande HD, Klein C, Klinger MR, Klinger LG | Neuropsychologia |
| [Neural correlates of anticipation and processing of performance feedback in social anxiety.](https://www.neurosynth.org/studies/25099708/) | Heitmann CY, Peterburs J, Mothes-Lasch M, Hallfarth MC, Bohme S, Miltner WH, Straube T | Human brain mapping |
| [Grey and white matter correlates of recent and remote autobiographical memory retrieval--insights from the dementias.](https://www.neurosynth.org/studies/25396740/) | Irish M, Hornberger M, El Wahsh S, Lam BY, Lah S, Miller L, Hsieh S, Hodges JR, Piguet O | PloS one |
| [Face-specific and domain-general characteristics of cortical responses during self-recognition.](https://www.neurosynth.org/studies/18501639/) | Sugiura M, Sassa Y, Jeong H, Horie K, Sato S, Kawashima R | NeuroImage |
| [Reduced cortical gray matter volume in male adolescents with substance and conduct problems.](https://www.neurosynth.org/studies/21592680/) | Dalwani M, Sakai JT, Mikulich-Gilbertson SK, Tanabe J, Raymond K, McWilliams SK, Thompson LL, Banich MT, Crowley TJ | Drug and alcohol dependence |
| [Altered task-based and resting-state amygdala functional connectivity following real-time fMRI amygdala neurofeedback training in major depressive disorder.](https://www.neurosynth.org/studies/29270356/) | Young KD, Siegle GJ, Misaki M, Zotev V, Phillips R, Drevets WC, Bodurka J | NeuroImage. Clinical |
| [Heightened activity in social reward networks is associated with adolescents' risky sexual behaviors.](https://www.neurosynth.org/studies/28755632/) | Eckstrand KL, Choukas-Bradley S, Mohanty A, Cross M, Allen NB, Silk JS, Jones NP, Forbes EE | Developmental cognitive neuroscience |
| [Impaired insight into illness and cognitive insight in schizophrenia spectrum disorders: resting state functional connectivity.](https://www.neurosynth.org/studies/25458571/) | Gerretsen P, Menon M, Mamo DC, Fervaha G, Remington G, Pollock BG, Graff-Guerrero A | Schizophrenia research |
| [Insular dysfunction within the salience network is associated with severity of symptoms and aberrant inter-network connectivity in major depressive disorder.](https://www.neurosynth.org/studies/24478665/) | Manoliu A, Meng C, Brandl F, Doll A, Tahmasian M, Scherr M, Schwerthoffer D, Zimmer C, Forstl H, Bauml J, Riedl V, Wohlschlager AM, Sorg C | Frontiers in human neuroscience |

**Appendix B. Meta-analysis result**

There are 12 clusters larger than 50 voxels, and 49 divided clusters(Table S2, Fiugre S1).

**
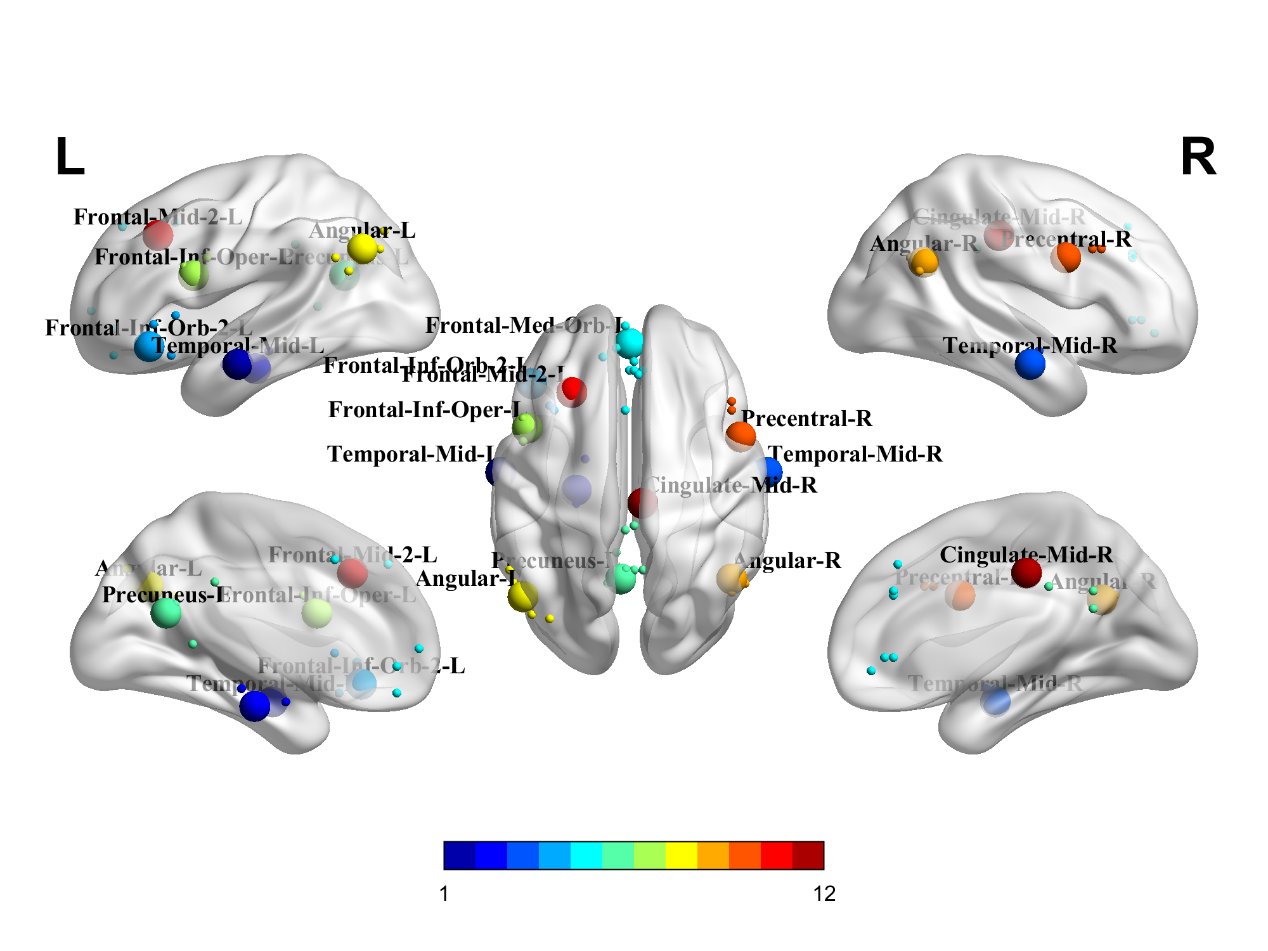
Figure S1.** Results of meta-analysis

| **Table S2.** Meta-analysis results from Neurosynth | | | | | | | |
| --- | --- | --- | --- | --- | --- | --- | --- |
| Cluster | Voxels | x | y | z | Intensity | AAL3 label | Region percent |
| 1 | 279 | -58 | -10 | -20 | 12.26 | Temporal_Mid_L |  |
|  | 261 | -58 | -10 | -20 | 12.26 | Temporal_Mid_L | 93.55% |
| 2 | 224 | -24 | -18 | -22 | 7.45 | None |  |
|  | 87 | -24 | -24 | -14 | 6.76 | Hippocampus_L | 38.84% |
|  | 68 | -20 | -4 | -20 | 6.08 | Amygdala_L | 30.36% |
| 3 | 175 | 62 | -10 | -20 | 6.76 | Temporal_Mid_R |  |
|  | 151 | 62 | -10 | -20 | 6.76 | Temporal_Mid_R | 86.29% |
|  | 24 | 56 | -6 | -14 | 5.39 | Temporal_Sup_R | 13.71% |
| 4 | 535 | -44 | 30 | -12 | 10.20 | Frontal_Inf_Orb_2_L |  |
|  | 187 | -44 | 30 | -12 | 10.20 | Frontal_Inf_Orb_2_L | 34.95% |
|  | 107 | -48 | 28 | -2 | 7.45 | Frontal_Inf_Tri_L | 20% |
|  | 104 | -34 | 18 | 2 | 7.45 | Insula_L | 19.44% |
|  | 84 | -36 | 20 | -16 | 7.45 | OFCpost_L | 15.70% |
|  | 38 | -46 | 30 | -14 | 8.14 | OFClat_L | 7.10% |
| 5 | 2736 | 0 | 48 | -10 | 12.94 | Frontal_Med_Orb_L |  |
|  | 954 | -2 | 56 | 4 | 12.26 | Frontal_Sup_Medial_L | 34.87% |
|  | 394 | -6 | 46 | -4 | 12.26 | ACC_pre_L | 14.40% |
|  | 270 | 4 | 34 | 42 | 4.70 | Frontal_Sup_Medial_R | 9.87% |
|  | 263 | 2 | 46 | -6 | 11.57 | Frontal_Med_Orb_R | 9.61% |
|  | 236 | 0 | 48 | -10 | 12.94 | Frontal_Med_Orb_L | 8.63% |
|  | 167 | 2 | 40 | 0 | 10.88 | ACC_pre_R | 6.10% |
|  | 88 | -12 | 42 | 42 | 6.08 | Frontal_Sup_2_L | 3.22% |
|  | 74 | -2 | 18 | 44 | 6.08 | Supp_Motor_Area_L | 2.70% |
|  | 51 |  |  |  |  | None | 1.86% |
|  | 47 | 6 | 36 | 28 | 6.08 | ACC_sup_R | 1.72% |
|  | 44 | 0 | 36 | 4 | 8.14 | ACC_sup_L | 1.61% |
|  | 40 | 2 | 36 | 30 | 6.08 | Cingulate_Mid_R | 1.46% |
|  | 40 | 2 | 36 | 0 | 7.45 | ACC_sub_R | 1.46% |
|  | 28 | 0 | 36 | 2 | 7.45 | ACC_sub_L | 1.02% |
|  | 23 | -4 | 46 | -16 | 6.08 | Rectus_L | 0.84% |
| 6 | 1268 | -4 | -58 | 20 | 13.63 | Precuneus_L |  |
|  | 588 | -4 | -58 | 20 | 13.63 | Precuneus_L | 46.37% |
|  | 167 | -2 | -54 | 24 | 13.63 | Cingulate_Post_L | 13.17% |
|  | 157 | 2 | -54 | 22 | 10.88 | Precuneus_R | 12.38% |
|  | 125 | -2 | -36 | 34 | 8.82 | Cingulate_Mid_L | 9.86% |
|  | 64 | 2 | -34 | 32 | 8.14 | Cingulate_Mid_R | 5.05% |
|  | 50 | -6 | -46 | 6 | 6.76 | Calcarine_L | 3.94% |
|  | 42 |  |  |  |  | None | 3.31% |
|  | 40 | -10 | -60 | 20 | 6.08 | Cuneus_L | 3.15% |
|  | 32 | 6 | -54 | 30 | 10.20 | Cingulate_Post_R | 2.52% |
| 7 | 410 | -46 | 10 | 20 | 10.20 | Frontal_Inf_Oper_L |  |
|  | 179 | -46 | 10 | 20 | 10.20 | Frontal_Inf_Oper_L | 43.66% |
|  | 113 | -48 | 14 | 24 | 8.14 | Frontal_Inf_Tri_L | 27.56% |
|  | 112 | -48 | 4 | 28 | 6.76 | Precentral_L | 27.32% |
| 8 | 734 | -48 | -66 | 32 | 15.01 | Angular_L |  |
|  | 503 | -48 | -66 | 32 | 15.01 | Angular_L | 68.53% |
|  | 78 | -36 | -76 | 40 | 6.08 | Parietal_Inf_L | 10.63% |
|  | 65 | -52 | -60 | 22 | 8.14 | Temporal_Mid_L | 8.86% |
|  | 55 | -44 | -74 | 32 | 6.76 | Occipital_Mid_L | 7.49% |
|  | 25 | -54 | -54 | 28 | 6.08 | SupraMarginal_L | 3.41% |
| 9 | 367 | 46 | -58 | 26 | 9.51 | Angular_R |  |
|  | 288 | 46 | -58 | 26 | 9.51 | Angular_R | 78.47% |
|  | 37 | 46 | -64 | 28 | 8.14 | Occipital_Mid_R | 10.08% |
|  | 26 | 52 | -60 | 22 | 5.39 | Temporal_Mid_R | 7.08% |
| 10 | 105 | 50 | 6 | 28 | 6.08 | Precentral_R |  |
|  | 40 | 46 | 22 | 32 | 6.08 | Frontal_Mid_2_R | 38.10% |
|  | 40 | 46 | 18 | 32 | 6.08 | Frontal_Inf_Oper_R | 38.10% |
|  | 24 | 50 | 6 | 28 | 6.08 | Precentral_R | 22.86% |
| 11 | 98 | -26 | 26 | 38 | 6.08 | Frontal_Mid_2_L |  |
|  | 74 | -26 | 26 | 38 | 6.08 | Frontal_Mid_2_L | 75.51% |
|  | 24 | -24 | 26 | 40 | 6.08 | Frontal_Sup_2_L | 24.49% |
| 12 | 53 | 6 | -24 | 38 | 6.08 | Cingulate_Mid_R |  |
|  | 46 | 6 | -24 | 38 | 6.08 | Cingulate_Mid_R | 86.79% |

**Appendix C. Graph theoretical analysis results**

| **Table S3.** Evaluation Metrics | | | | | | | |
| --- | --- | --- | --- | --- | --- | --- | --- |
|  | | DPD | | HC | | Average / Total | |
| Support |  | 17 |  | 9 |  | 26 |  |
| Accuracy |  | 0.885 |  | 0.885 |  | 0.885 |  |
| Precision (Positive Predictive Value) |  | 0.938 |  | 0.800 |  | 0.890 |  |
| Recall (True Positive Rate) |  | 0.882 |  | 0.889 |  | 0.885 |  |
| False Positive Rate |  | 0.111 |  | 0.118 |  | 0.114 |  |
| False Discovery Rate |  | 0.063 |  | 0.200 |  | 0.131 |  |
| F1 Score |  | 0.909 |  | 0.842 |  | 0.886 |  |
| Matthews Correlation Coefficient |  | 0.754 |  | 0.754 |  | 0.754 |  |
| Area Under Curve (AUC) |  | 0.928 |  | 0.928 |  | 0.928 |  |
| Negative Predictive Value |  | 0.800 |  | 0.938 |  | 0.869 |  |
| True Negative Rate |  | 0.889 |  | 0.882 |  | 0.886 |  |
| False Negative Rate |  | 0.118 |  | 0.111 |  | 0.114 |  |
| False Omission Rate |  | 0.200 |  | 0.063 |  | 0.131 |  |
| Threat Score |  | 3.750 |  | 1.600 |  | 2.675 |  |
| Statistical Parity |  | 0.615 |  | 0.385 |  | 1.000 |  |
| *Note.*  All metrics are calculated for every class against all other classes. | | | | | | | |
